# Perceived Sensitivity of Sensor-Based Digital Health Data: Qualitative Interview Study

**DOI:** 10.2196/78788

**Published:** 2026-07-08

**Authors:** Christine Deeney, Anika Sonig, Meghan E Hurley, Birkan Tunç, Eric A Storch, John D Herrington, Jennifer Blumenthal-Barby, Kristin Kostick-Quenet

**Affiliations:** 1 Center for Medical Ethics and Health Policy, Baylor College of Medicine, One Baylor Plaza, Suite 310D, Houston, TX, 77030, United States, 1 713-798-4951; 2 Department of Child and Adolescent Psychiatry and Behavioral Services, Children's Hospital of Philadelphia, Philadelphia, PA, United States; 3 Department of Psychiatry, Perelman School of Medicine, University of Pennsylvania, Philadelphia, PA, United States; 4 Menninger Department of Psychiatry and Behavioral Services, Baylor College of Medicine, Houston, TX, United States

**Keywords:** mobile health, mHealth, trust, data sharing, data protection, data privacy, transparency, digital phenotyping, affective computing

## Abstract

**Background:**

Digital health tools are increasingly used in mental health care to passively collect patient data and analyze health status outside of clinical settings. While technologies such as digital phenotyping, affective computing, and computational behavioral analysis offer new insights into symptom manifestation in daily life, they generate large volumes of potentially sensitive data that raise significant data privacy concerns, requiring high levels of patient awareness and consent. Empirical research is lacking on stakeholder understandings toward the sensitivity of these data and expectations for data stewardship, perspectives that are critical for developing robust informed consent and data protection policies for digital health data use.

**Objective:**

This study aimed to explore key stakeholder perspectives on the sensitivity of computer perception (CP) data, trust in existing data protections, willingness to share CP data externally, and desire for transparency of CP data transactions outside of the clinical space.

**Methods:**

As part of a larger, multisite study, we conducted qualitative interviews (n=40) via Zoom (Zoom Communications, Inc) with 20 adolescents (aged 12‐17 years) familiar with CP tools and their caregivers (n=20). Interviews consisted of a series of open-ended questions regarding stakeholders’ perspectives on privacy, data security, and the use and exchange of CP data. We developed a qualitative codebook to identify and label thematic patterns in responses to questions addressing the topics above, using thematic content analysis to identify themes inductively. Each interview was coded by merging work from at least two separate coders, and several team members contributed to qualitative analysis.

**Results:**

Most adolescents and caregivers viewed CP data as highly sensitive and expressed a reluctance to share these data beyond their clinical teams. While many participants expressed trust in existing data protections to protect CP data, they often misunderstood or overestimated the extent of protections to safeguard CP data.

**Conclusions:**

Our findings underscore the critical need for clear and effective patient communication and education about the risks, benefits, and protections associated with CP data through informed consent protocols. To promote greater transparency, understanding, and trust, we recommend 5 strategies: educating patients about data protection; studying secondary data exchange and reidentification risks; strengthening transparency regulations; improving data traceability mechanisms, such as distributed ledger technologies, to enhance data traceability and auditability; and adopting dynamic consent models.

## Introduction

Mental health care is undergoing rapid change as researchers and clinicians adopt digital tools and mHealth (mobile health) apps to support diagnosis and monitoring. Approaches such as digital phenotyping [1-5], affective computing [6,7], and computational behavioral analysis [8,9] involve algorithmic analysis of continuously and passively collected data from patients outside of clinical settings in an attempt to offer more ecologically valid insights into how symptoms manifest in daily life. We refer to these approaches as computer perception (CP) technologies, a term that builds on computer vision but reflects a broader set of sensing methods, including voice, movement, location, and physiological signals (eg, heart rate and skin conductance). Despite their nuanced distinctions [5], these CP approaches generate large volumes of potentially sensitive data to enable various forms of clinical inference, particularly when triangulated and used alongside remotely administered self-report measures (“ecological momentary assessments”), often via mHealth platforms. Given their promise for data-centric and value-based care, CP technologies are increasingly applied across psychiatry and other areas of medicine (eg, cardiology [10], pain management [11], maternal health [12], and asthma [13]) that benefit from insight into behavioral patterns outside the clinic.

At the same time, CP tools also introduce significant ethical and privacy concerns, necessitating robust approaches to informed consent and data protection that directly respond to how individuals understand the sensitivity of these data and how they are—or should be—protected. A major challenge in digital health is the misalignment between stakeholder expectations and actual data governance practices [14-16]. Individuals may assume these data are safeguarded under existing health privacy laws, such as the HIPAA (Health Insurance Portability and Accountability Act), even when these data fall outside formal regulatory coverage [17-19]. Prior work shows that when perceived safeguards prove illusory, this misalignment can erode trust, reduce willingness to share data, and compromise sustained engagement with digital health tools [20].

This paper builds on this literature by presenting results from in-depth interviews exploring stakeholder understandings of the sensitivity of CP data, trust in existing data protections, willingness to share CP data for clinical or research purposes, and preferences for transparency around CP data transactions outside the clinical space. Although our empirical data were collected in the United States and reference US health privacy frameworks, the ethical and practical challenges we examine are not unique to North America. Across jurisdictions, rapid growth in digital health and sensing technologies has outpaced public understanding of how personal data is governed, shared, and reused. Even under the European Union’s General Data Protection Regulation (GDPR), which provides broader formal protections for certain categories of personal data, enforcement gaps, limited transparency, and user misunderstandings persist, particularly for passively collected behavioral and biometric data [21].

Many CP data are not directly clinical in nature. Unlike traditional medical data, CP data may be collected for clinical purposes while remaining usable for nonclinical applications, such as marketing or profiling [19,22]. Importantly, CP data often fall between established categories of personal data, combining elements of identity data (eg, location, voice, facial features, and device identifiers) and clinical data (eg, inferred symptoms or functional states), even when they are not explicitly diagnostic on their own. Public perspectives on digital health data indicate that users may not perceive their self-tracked health information with the same sensitivity as traditional personal health information (PHI), despite the fact that these data can reveal sensitive information about a person’s health or reproductive status [19].

As these data are not always classified as PHI, these “nontraditional health” (NTH) data often receive weaker legal protections, particularly in jurisdictions where health privacy laws apply narrowly to clinical records and covered entities. In the United States, for example, many CP data fall outside the scope of HIPAA, whereas in other jurisdictions, such as the European Union, broader data protection frameworks such as the GDPR may apply, at least in principle, to biometric and behavioral data. However, this regulatory ambiguity creates gaps in transparency, accountability, and user awareness [23,24]. Additionally, public misunderstandings regarding GDPR’s scope in relation to biometric or behavioral tracking data contribute to ongoing challenges in ensuring informed consent and ethical data management [25].

We argue that this misalignment reflects a more fundamental lack of computational mechanisms, alongside supportive regulatory frameworks, that enable transparent tracking of patient and consumer data. This lack of visibility into how data moves across clinical, research, and commercial contexts perpetuates misunderstandings, power imbalances, and trust gaps between individuals, institutions, and technology systems, leading to collective vulnerabilities in privacy, autonomy, and equitable access to digital health benefits.

These ambiguities leave NTH data vulnerable to unwanted or undisclosed forms of data access and exchange by third-party data controllers (eg, large technology companies), particularly given that many mHealth apps lack adequate data security and confidentiality assurances [26]. Further, because many CP data are generated in the context of research-industry partnerships, uncertainties persist around what may be done with these data, to which entities control or claim ownership rights, where data are stored (eg, hospital vs corporate servers), and who holds decision-making authority over data access and exchange (eg, researchers, companies, or data brokers). A growing body of research suggests that public trust in data protection measures varies by institutional affiliation, with greater skepticism toward commercial data handlers [27].

Such concerns extend across data types, ranging from PHI to NTH data, collected within health care [19,22,28,29] as well as outside of health care, including browser history and consumer behavior data used for targeted marketing and personalization [29,30]. Extensive empirical literature documents public concerns about data privacy, transparency, and control and a desire for greater authority over what personal information is collected and who has access to it [27,31-35]. While numerous studies have examined perspectives on privacy, security, and confidentiality in mHealth apps [36-38], and others have explored attitudes toward CP technologies in relation to acceptability, adoption of, and adherence [37-45], few specifically investigate how individuals understand the handling of CP data after collection or how these understandings align with the realities of health information exchange (HIE) in the United States and elsewhere.

Patients and research participants may assume these data constitute PHI when collected in clinical or research settings, leading to misinterpretations of the degree to which these data are protected under data privacy laws—much as consumers often assume their personal data are protected by default on websites or mobile apps without reading the terms of service or privacy policies [46-48]. Such misinterpretations are compounded by limited transparency about the benefits and risks of collecting CP data at the time of consent, as computational analysis capabilities continue to expand.

Examining alignment or misalignment in understandings about the nature and governance of CP data is therefore critical for developing patient education and informed consent protocols that clearly articulate potential benefits and risks. In this paper, we present findings from a qualitative investigation of adolescents’ and caregivers’ perspectives on sharing passively collected CP data. Our study aimed to explore key stakeholder perspectives on: (1) the perceived sensitivity of CP data; (2) trust in existing data protections governing CP data; (3) willingness to share CP data for clinical or research purposes; and (4) preferences for transparency around CP data practices and transactions—with the goal of informing more robust informed consent protocols and data governance recommendations for CP and related digital health technologies. We focus on CP data collected in settings where research and clinical care increasingly overlap, such as clinical trials embedded in health care systems, validation studies for tools intended for clinical deployment, and digital health platforms developed through academic-industry partnerships. While research and clinical contexts are governed by distinct ethical and regulatory standards, participants’ expectations about data protection and use often span, or fail to distinguish between, these domains. We conclude by offering targeted recommendations to improve transparency and informed consent practices surrounding the collection and exchange of CP and other digital health data.

## Methods

### Study Design

This research aimed to explore ethical considerations for translating CP technologies into clinical care. Through in-depth, semistructured interviews, we explored perspectives on benefits, risks, and concerns around the integration of CP technologies, which also involved a supplementary exploration of specific concerns around privacy, data security, and perspectives toward the use and exchange of CP data.

### Participants

As part of a multisite study funded by the National Center for Advancing Translational Sciences (R01TR004243), we recruited 40 participants between January 2023 and August 2023. Participants were recruited from a sister research study (R01MH125958) conducted within a clinical research setting, focused on validating CP tools designed to quantify objective digital biobehavioral markers of socioemotional functioning. Participants in our sister study were recruited from a clinical care setting in a large academic medical center using word of mouth. As part of that study, CP data were collected in a laboratory setting for research purposes with the stated goal of informing future clinical applications. As such, participant perspectives reflect experiences situated at the boundary of research and clinical care. Our choice to recruit from this sister study was also to ensure familiarity with and exposure to the collection of CP data, to enhance the ecological validity of our findings. Participants included adolescents (n=20) aged 12‐17 years and their caregivers (typically biological parents, see Table 1). The adolescents had previously received neurodevelopmental (ie, autism) and/or psychiatric diagnoses of autism, anxiety, or depression and potentially diagnoses that often accompany these (eg, attention deficit/hyperactivity disorder or obsessive compulsive disorder). Diagnostic presentations for all adolescents were confirmed through the administration of standardized clinician interviews. Participants were told about this study by a research coordinator from our sister study. Individuals who expressed interest in participating were referred to this study by the sister study’s coordinator and then contacted by a research assistant (RA) via phone or email to schedule a virtual interview. Our response rate was approximately 63% for patients and caregivers (63 people were contacted and 40 people agreed to participate).

**Table 1.** Demographics for interviewed adolescents and caregivers. Values may not total to 100% due to categorical overlap (eg, comorbidities). Frequencies and percentages reflect only reported information and are not intended to suggest any level of statistical significance.

	Adolescents (n=20, 50%)	Caregivers (n=20, 50%)	Total (N=40, 100%)
Sex, n (%)			
Male	12 (30)	2 (5)	14 (35)
Female	8 (20)	18 (45)	26 (65)
Race or ethnicity, n (%)			
American Indian or Alaska Native	0 (0)	1 (3)	1 (3)
Asian	1 (3)	1 (3)	2 (5)
Black or African American	5 (13)	4 (10)	9 (23)
Native Hawaiian or Other Pacific Islander	0 (0)	0 (0)	0 (0)
White	17 (43)	15 (37)	32 (80)
Hispanic or Latino	4 (10)	2 (5)	6 (15)
Not Hispanic or Latino	16 (40)	18 (45)	34 (85)
Marital status, n (%)			
Married and living with spouse	—^a^	13 (65)	13 (33)
Widowed	—	1 (5)	1 (3)
Divorced	—	4 (20)	4 (10)
Separated	—	1 (5)	1 (3)
Never married	—	1 (5)	1 (3)
Education, n (%)			
High school only or less	—	0 (0)	0 (0)
Trade school or associate’s degree	—	2 (10)	2 (5)
Bachelor’s degree	—	10 (50)	10 (25)
Master’s degree	—	4 (20)	4 (10)
Doctoral degree	—	4 (20)	4 (10)
Parental status, n (%)			
Biological parent	—	18 (90)	18 (45)
Stepparent	—	0 (0)	0 (0)
Adoptive parent	—	2 (10)	2 (5)
Diagnosed condition, n (%)			
OCD^b^	4 (20)	—	4 (10)
Autism	5 (25)	—	5 (13)
ADHD^c^	3 (15)	—	3 (8)
Anxiety	4 (20; 1 self-reported)	—	4 (10)
Tourette	1 (5)	—	1 (3)
No clinical diagnosis or symptoms	9 (45)	—	9 (45)
Age, mean (SD)			
Average age	14.9 (2.2)	48.3 (6.4)	—

a Dashes indicate categories for which n=0 (0%).

b OCD: obsessive-compulsive disorder.

c ADHD: attention-deficit hyperactivity disorder.

### Data Collection

We developed separate interview guides for adolescents and their caregivers (MultimediaAppendices 1  2), exploring the same constructs across both groups with appropriate variations in wording. Guides were informed by prior work exploring user perspectives in data privacy and governance [14,15,17,19,20,22,27,29-38] and with the guidance of experienced bioethicists, child mental health experts, and computational scientists who develop these algorithms. Constructs included data security and privacy issues, regarding perceived sensitivity of this type of data and data collection, beliefs about the potential for unintended uses and current data protections, thoughts on secondary usage of their data and access authorization, and transparency needs or preferences of their personal data and its transactions or security processes. Initial drafts of the interview guides were piloted with two psychologists specializing in adolescent mental health, resulting in minor clarifications in wording. Interviews were conducted by RAs (authors CD, AS, and MEH) with extensive training in qualitative interviewing and analysis. Interviews were held via a secure videoconferencing platform (Zoom for Healthcare [Zoom Communications, Inc]) and lasted an average of ≈45 minutes. Before beginning the interview, each participant was presented with a 7-minute primer video (Multimedia Appendix 3) explaining the nature and intended use of CP data in clinical research and health care (ie, a basic, 8th grade-level understanding of how CP technologies collect and analyze data and how they might be used during a clinical encounter), including the different types of CP technologies available and the types of data they collect (eg, different types of wearables, smart devices, and sensors that stakeholders would likely be familiar with and that can collect vocal, facial, accelerometry, GPS, heart rate data, and more). The primer video was developed to ensure informed responses from patients and caregivers who are likely to be less familiar with this suite of technologies. Additionally, the RA stopped the video at key predesignated intervals (eg, after the original explanation of how CP collects and analyzes patient data, after providing an example of what the integration of these tools into a traditional clinical assessment could look like, etc) to assess understanding and elicit clarifying questions from participants to ensure comprehension. Recruitment continued until we observed significantly diminished novel informational returns from each subsequent interview (“saturation”). Operationally, research team members reviewed transcripts to identify novel insights not present in prior interviews. Saturation was determined when successive interviews within a stakeholder group yielded no new insights, which occurred in around 20 interviews per stakeholder group.

### Ethical Considerations

This study was reviewed and approved by the Baylor College of Medicine Institutional Review Board (H-52233), which also waived a requirement for written consent due to research procedures involving minimal risk to participating stakeholders; thus, participants provided verbal consent. Minors provided assent with parental consent. Identifying participant information was stored in a password-protected system with 2-factor authentication behind a university firewall. All results are reported in the aggregate and are not linked to identifiable participants. Each participant received a US $50 gift card as compensation for their time.

### Data Analysis

**Figure 1.**
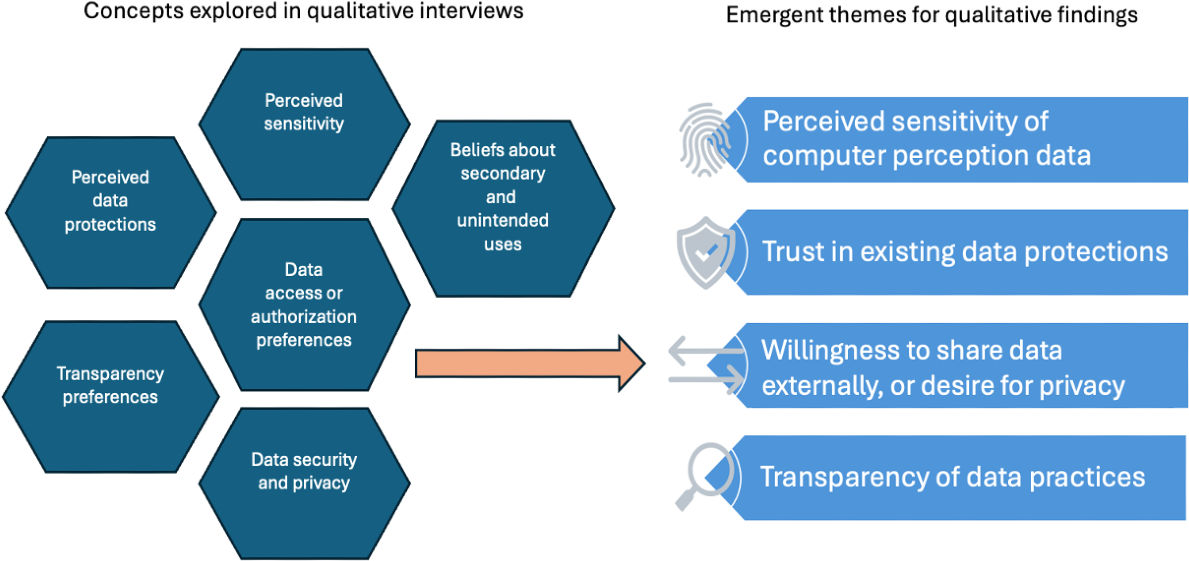
Transformation of qualitative inquiry domains into thematic findings.

Interviews were audio-recorded, transcribed verbatim, and analyzed using MAXQDA (VERBI GmbH) software. Led by a qualitative methods expert, team members developed a qualitative codebook to identify and label thematic patterns in adolescent and caregiver responses to questions addressing the topics above. Codebook development followed a hybrid inductive–deductive approach. We formed an initial set of broad analytic domains informed by prior literature on digital health ethics, data governance, trust, privacy, and informed consent [19,23-27,29-38], which served as sensitizing concepts rather than fixed categories. Within these domains, codes were developed inductively through our familiarity with the interview content (all team members listened to all interview recordings) and a close review of a subset of transcripts to capture emergent concepts. Codes and definitions were iteratively refined in collaborative team meetings, and subsequently used to identify patterns in the data using thematic analysis [49]. Specifically, outputs for each code were further analyzed by progressively abstracting relevant quotes, an inductive process that entails reading every quotation to which a given code was attributed, paraphrasing each quotation (primary abstraction), and further identifying which constructs were addressed by each quotation (secondary abstraction). These constructs mapped onto a core set of thematic findings across four emergent domains (see Figure 1). Each of the concepts in these four domains was further categorized along a scale of “high,” “medium,” or “low” (eg, “high” need for transparency) to examine relationships between beliefs and perspectives, informational needs and preferences, and trust. These designations reflect consensus-based qualitative judgments by the research team, grounded in close analysis of coded excerpts, rather than participant ratings or a formal scale. Each interview was coded by merging work from at least two separate coders to reduce interpretability bias and enhance reliability. To ensure the validity of our findings, all abstractions were validated by at least one other member of the research team before calculating thematic frequencies to characterize stakeholders’ responses and obtain a sense of the proportion of stakeholders raising or discussing each concern/theme. In rare cases where abstractions reflected conflicting interpretations, members of our research team met to reach consensus. Frequencies were used as a descriptive aid to convey the relative salience of perspectives across participants, consistent with pattern-based thematic analysis, and were not intended to suggest statistical significance or quantitative generalizability [49]. Frequencies account for participants who expressed perspectives that were ambiguous or context-dependent (eg, perceiving CP data to be highly sensitive in some situations but not in others) and were calculated when respondents expressed views across the high, moderate, and low categories. Frequencies and percentages reflect only reported information and therefore do not always sum to 100%, as some respondents did not express perspectives across all themes.

## Results

### Overview

The results are organized around four analytic domains identified through thematic analysis of transcripts from 20 adolescents and 20 caregivers (total N=40; see Table 1). These domains include the following: (1) perceived sensitivity of CP data; (2) trust in existing data protections; (3) willingness to share data externally and desire for privacy; and (4) transparency of data practices (see Table 2). Within each domain, we describe the range of perspectives expressed by participants and use qualitative descriptors (eg, high, moderate, and low) to summarize relative emphasis and salience, illustrated with representative quotations (eg, higher to lower perceived sensitivity and higher to lower trust in existing data protections). Frequencies and percentages of expressed views across these domains are presented in Table 2, intended to aid interpretive comparison between and across groups. Taken together, our results indicate that most caregivers and adolescents view CP data as moderately to highly sensitive, are reluctant to share these data beyond their clinical teams, and expressed a desire to have visibility into or control over both primary and secondary uses of their data. Notably, adolescents expressed higher trust in existing data protections compared to caregivers. Most caregivers shared that they were less willing to share data externally compared to adolescents. The following sections elaborate on and summarize these findings, complemented by illustrative quotes.

**Table 2.** Frequencies and percentages of expressed views across thematic domains.

	Adolescents, n (%)	Caregivers, n (%)	Total, n (%)
Perceived sensitivity of CP^a^ data			
High (only)	—^b^	1 (5)	1 (3)
Moderate to high	11 (55)	12 (60)	23 (58)
Moderate (only)	4 (20)	1 (5)	5 (13)
Moderate to low	5 (25)	1 (5)	6 (15)
Ambivalent (both high+low)	1 (5)	1 (5)	2 (5)
Significant ambivalence (high+ moderate+low)^c^	2 (10)	4 (20)	6 (15)
Low (only)	—	—	—
Trust in existing data protections			
High (only)	9 (45)	5 (25)	14 (35)
Moderate to high	3 (15)	3 (15)	6 (15)
Moderate (only)	2 (10)	6 (30)	8 (20)
Moderate to low	—	—	—
Ambivalent (both high+low)	—	—	—
Low (only)	—	—	—
Willingness to share CP data			
High (only)	—	—	—
Moderate to high	—	—	—
Moderate (only)	—	1 (5)	1 (3)
Moderate to low	9 (45)	18 (90)	27 (68)
Ambivalent (both high+low)	—	—	—
Significant ambivalence (high+ moderate+low)^c^	4 (20)	—	4 (10)
Low (only)	—	—	—
Desire for transparency in use or secondary use of CP data			
High (only)	11 (55)	12 (60)	23 (58)
Moderate to high	3 (15)	3 (15)	6 (15)
Moderate (only)	—	1 (5)	1 (3)
Moderate to low	—	—	—
Ambivalent (both high+low)	—	—	—
Low (only)	—	—	—

a CP: computer perception.

b Dashes throughout the table represent areas where no participant expressed perspectives that fall into those categories (eg, no adolescents or caregivers viewed computer perception data to be “low sensitivity”).

c This category was only present for these 2 themes but represents significant ambivalence among certain participants who expressed views across each level.

### Perceived Sensitivity of Passive Continuous Monitoring Data

Most participants (15 adolescents and 14 caregivers) perceived CP data to be highly sensitive. Participants commonly understood CP data as revealing intimate aspects of identity, emotion, and daily life, leading many to view continuous monitoring as intrusive even when collected for clinical purposes. Some characterized it as an “invasion of privacy” (A16) because such data capture the “emotions and feelings of a person” (A16) or “who you are on the inside” (CG03; see Table 3). Some caregivers characterized continuous monitoring as “unnerving” (CG08) or “debilitating” (CG03), or likened it to “big brother” surveillance (CG19). More than half of all participants expressed a desire to pause or disable monitoring during moments they considered private. These moments included private conversations, conflicts with family members, funerals, intimate situations, or emotionally vulnerable situations. Both stakeholder groups expressed a view that the potential negative impacts of collecting CP data may be especially pronounced in the context of physical and socioemotional changes associated with adolescence (eg, “where it would affect [their] self-image” CG07).

By contrast, a smaller number of participants (5 adolescents and 1 caregiver) viewed CP data as less sensitive, with one adolescent stating that it felt “sort of like wearing a Fitbit” (A07), particularly when perceived clinical benefits were salient (as noted by 6 adolescents and 9 caregivers).

Some stakeholders (3 adolescents and 5 caregivers) expressed ambivalence. For example, one caregiver shared that while the data itself did not seem invasive, since “our watches collect and our phones collect this kind of stuff all the time” (CG01), they would nevertheless be bothered if the data were shared with third parties (eg, insurance companies and future employers) without their knowledge (see also Willingness to Share CP Data section below).

**Table 3.** Quotes illustrating varying levels of perceived sensitivity of CP^a^ data.

Perceived sensitivity: stated rationales	Adolescent quote	Caregiver quote
Highly sensitive		
CP data deserves greater protection than biological data	“It is a little bit more sensitive because it feels a lot more with the emotions and feelings of a person. So I do think they should be treated with a little bit of extra care.” A16	“We need to have more protection [for CP data]. Because it’s just different information. That’s the voice, the facial expressions, again, that it’s not going to be used for wrong purposes.” CG16
CP data deserves the same high legal protections as other medical data	“It should be treated equal, because all medical information, unless you actually disclose it to somebody, it’s not supposed to be known.” A01	“Probably about the same [amount of protection]... Certainly not less.” CG01
CP captures highly sensitive (personal) information: emotions, facial expressions, etc	“I think some people would maybe be reluctant to do things like this... Maybe people are afraid that something…may get exposed or… don’t want someone examining their facial expressions [or] they don’t want other people to know about something [a condition] they may or may not have.” A01	“The physician has access to your naked body. But with emotions it’s like having access to the naked naked because it’s really who you are on the inside too. Things that you are unable to express. A machine that would be able to intimately know where you are, it’s absolutely debilitating, it’s scary.” CG03
Passive continuous data collection is invasive and unsettling	“I wouldn’t necessarily want it to monitor everything that I say. I would think that would be a bit invasive.” A02 "It feels kind of invasive because I feel like it would just depend on the situation. You don’t know, maybe there’s a reason you aren’t telling people about it and if they know without asking...it just seems invasive sometimes.” A14	“So her DNA test says…she has a 40% higher chance than most people of developing, in the future, age related macular degeneration. That was helpful. So she takes lutein and ocuvite because it’s progressive through time. But that was very easy to do. It was private. Blood test. She knows. I know. Her doctor knows, but it’s not as invasive as what you’re proposing.” CG07
CP data collection is especially sensitive for adolescents	“If I was getting in a fight with my siblings or stuff like that, maybe I wouldn’t want that to be recorded.” A13	“When they hit high school. It gets very difficult. You’ve got all of those other complexities, the peers and society, I’m trying to figure out how I fit. It’s really complex. When you’re talking personal intrusiveness, I don’t know. I’m a little sketchy on that.” CG11
Moderately sensitive		
CP is invasive but justified by clinical benefits	“It’s a little invasive. I personally wouldn’t want my doctor to see like every single emotion and feeling that I’m having throughout the day. But at the same time, if there’s someone who’s unable to tell their feelings or emotions or what they’re feeling properly, then it could be great help. It’s helpful at the same time.” A04	“If we come to the doctor and they explain, ‘Hey, this is going to be better to help me, help you,’ then sure, but unless we specifically come with a very specific issue... That’s probably overkill. They deserve some privacy.” CG04
Prefer ability to turn off continuous data collection in certain circumstances	“I feel actually most places I would probably just have it turned off because I don’t like the idea of people knowing where I am at all times in case that data gets out… That just seems like a weird thing to me.” A07 “When I’m going to the restroom or something like that.” A17 “Probably when I’m taking a shower or something.” A05 “Probably just my sleep.” A04	“What if she wants to get intimate with her partner or to take a bath or she’s having a really hard bowel movement that day. Those are certain things that might raise your heart rate that you don’t necessarily want anybody to know about. It’s very personal and doesn’t necessarily need to be part of her diagnosis process... I think there should definitely be times you’re allowed to take it off.” CG05
CP data is more vulnerable to misinterpretation (needs context, can be subjective, etc)	“I guess the eye tracking and the listening to my voice**…**it’s all being processed by maybe someone else or AI or something like that. And maybe someone else interprets what you’re feeling differently than what you are actually feeling. Maybe you’re trying to hide something and they just don’t see it.” A02	“I just worry that it could be wrong because sometimes computers don’t know everything. They’re not human. And it would be messed up for it to say, oh, well this person is feeling this and that person is not feeling that. It could mess up something. Oh, your child’s feeling sad, to the point where they want to kill themselves. And [your kid is] like, ‘No, that’s not what I feel.’ So I just worry about the error rate.” CG08
Less sensitive		
No strong desire to turn off continuous monitoring in certain situations	“I mean there’s nothing that’s insanely private. I mean, [there aren’t any moments where I would not want it to collect data in the moment], not that I can think of off the top of my head at least.” A12	“Again, without being in that moment, no... I can’t think of a thing where I would want total and utter privacy.” CG10
Views CP as having a similar (low) sensitivity as other wearable or smartphone data	“I don’t have that huge of a problem with it. I think it’s sort of like wearing a Fitbit where it counts your steps and things like that. My only concern would be privacy, being shared with other people.” A07	“Our watches collect and our phones collect this kind of stuff all the time. I think that if that’s being transmitted to your insurance company or potential life insurance or disability insurance companies and they’re going to rate you based on things like that, I guess that would probably bother me to some extent. But you don’t want to get paranoid about it, you just want to be realistic about it.” CG01

a CP: computer perception.

### Trust in Existing Data Protections

Most participants (14 adolescents and 14 caregivers) expressed moderate to high trust in existing data protections governing CP data (see Table 4). This trust was largely grounded in confidence in clinicians and health care institutions, often accompanied by assumptions that CP data are protected under established health privacy laws. Several adolescents stated they had no concerns about CP data being shared beyond their doctor (eg, A11 and A13), while others emphasized that clinicians “are there to help you” and therefore could be trusted with sensitive data (A16). Some caregivers explicitly assumed that CP data were protected under HIPAA, stating that they “would expect the same type of privacy laws to apply” (CG03). This subset expressed more cautious trust, noting that while they had “no major concerns,” they still wanted reassurance that data protections were “following HIPAA” (CG05). Notably, no respondents expressed low or no trust in existing data protections.

**Table 4.** Quotes illustrating varying levels of trust in existing data protections.

Trust: stated rationales	Adolescent quote	Caregiver quote
High trust		
Few, if any, concerns or perceived harm from sharing CP^a^ data	“I think it depends on who it would be shared to, but I don’t think for the majority I would have a problem with that being information being shared.” A02	“I’m not sure what kind of problems could really arise from that [information collected potentially be shared with people other than their doctor].” CG09
Less concerned about unauthorized and harmful external data sharing because they trust or expect that it is kept safe/secure	“With every bit of information being locked down with multiple encrypted passwords, and everything’s double encrypted, I have no concern.” A09	“I know it’s a very confidential doctor patient kind of relationship so there’s no harm to my child’s reputation.” CG20
Expects/assumes HIPAA^b^ already protects this type of user-generated data	“I guess it really depends on who it’s getting shared to, but I guess it would be the same as what HIPAA is for. It would just be sent to the people that would be able to act upon it super fast...” A19	“I feel like it would be a part of their health information, so it would be private. I’m not any more worried about the technology specifically as opposed to overall somebody knowing he has an anxiety or OCD.” CG18
Moderate trust		
Wants to be reassured of HIPAA protection	“What I would need though as a very firm understanding of the boundaries of the utilization of that data because HIPAA’s a pretty big thing... I would want to know could anything in this data be not protected, why and how.” CG12	“I want to know that it’s HIPAA approved and although there’s always a risk for a leak data because it’s not a perfect world and there’s a lot of hackers out there, just knowing that it’s as safe as possible.” CG05
Limited trust of technology developers to respect privacy (fear of technology)	“Even though you said that it wouldn’t, I feel like I would always be nervous that it is tracking the things that I’m saying and what I’m actually doing, like even though it says it isn’t, I feel like that would just be a fear.” A14	“Right, the privacy aspect of it. Is it actually collecting texts or voicemails or recording conversations? We’re always questioning whether our Alexa is actually listening to us.” CG14
Low trust	—^c^	—

a CP: computer perception.

b HIPPA: Health Insurance Portability and Accountability Act.

c Not applicable.

### Willingness to Share CP Data

Most participants were reluctant to share CP data beyond their immediate clinical teams. Reluctance to share reflected concerns about privacy, loss of control, and potential misuse of data, particularly among caregivers responsible for their children’s information. Participants frequently emphasized ownership and control, with one adolescent stating, “It’s my information, keep it that way” (A05). Caregivers expressed near-universal hesitancy about external sharing, particularly with commercial entities. Notably, twice as many caregivers (n=18, 90%) expressed this reluctance as adolescents (n=9, 45%) (Table 2). Their rationales (see Table 5) centered around a common view of these data as invasive and sensitive. At the same time, some participants expressed ambivalence or conditional willingness, acknowledging that CP data could be “helpful” (A16) or valuable for research while simultaneously asserting that such data “should be mine and my doctor’s, and that’s it” (A07). Some adolescents and caregivers stated a preference to be able to vet or control what information their doctors could access. A small number expressed resignation or apathy, suggesting that data sharing “does not make much difference” given the broader data ecosystem (A02).

**Table 5.** Quotes illustrating varying willingness to share CP^a^ data.

Desire for privacy and willingness to share: stated rationales	Adolescent quote	Caregiver quote
Most willing to share		
Positive attitudes toward secondary uses	“I think it’s probably going to be really helpful for future researchers if they can better whatever they’re trying to research and figure something out that could help a lot of people, then I’m all for it.” A16	—^b^
Moderately willing to share		
Secondary uses must come with stipulations (informed consent, intended use, reputation, and compensation)	“Yes (I would be willing if I knew) just what they’re using it for, how long they’re going to hold onto it, who gets to see it and is the company or whatever who’s accessing the data from a reputable source.” A04	“I’m comfortable with it as long as, if there is a way to let the participants know that, ‘Hey, there’s a chance that your information is going to be used in another study.’ And identify that study.” CG04
Willing to share if given control over data-sharing decisions	“I would be more willing to share if I have complete control over it.” A03	“If I have the ability to say no, I might be a little more open-minded about who wants to see it for why. But again, I would need to have that information.” CG02
CP data should be kept private, on a need-to-know basis with medical personnel (and treated like other medical data—not shared widely)	“I do think they should be kept private for a good amount... Not everybody has to see it except for my doctor and other people who me, my doctor, give authorized access to. But other than that should be pretty private.” A16	“I think that it should probably not go to future employers or colleges, it’s personal information. And I think too that now in this day and age… [data] is it sold somewhere to advertisers to then manipulate you? I mean, you can get paranoid about it, but I think that there is some level of that concern about what happens to that data.” CG01
Reluctant to share CP data when it oversteps relationship boundaries	“I feel like [I would] maybe [be worried about sharing this data with] my parents, I don’t know. If I’m trying to deal with something and I don’t want them on my back about it, I feel like I wouldn’t want them to know.” A14	“I could see that with the younger population. But then again, we have that crossover when they get to be young adults. It’s so intrusive and you would probably produce battles that you wouldn’t really want… there’s a certain level of that’s his right to not talk about it. It really sucks when I don’t know…But you have to really be careful on when they get older, there’s more permission that they have to give. It’s been hard to balance that…” CG11
Prefer to vet what doctors can see	“I think I’d have to know that it would show me the results first and then ask if I’m okay with sending it to the doctor, instead of just sending it to the doctor first and then the doctor telling the things. You get what I’m saying?” A07	“Can the doctor themselves go in and listen to a conversation because I wouldn’t like that. Now, if the data’s being analyzed in a way that the doctor doesn’t have access to and it’s shooting off reports, I’m far less troubled by that.**”** CG12
Least willing to share		
Would “not” influence willingness to share because control/privacy does not seem feasible, or they are not interested	“I don’t think it [sharing] would make much of a difference. I mean, there’s some stuff that’s probably already out there... But if it was something that really influences my health or just my mental wellbeing, if it really matters that much to me, I would probably care about it. But just in general, probably not.” A02	“I just don’t see that there’s really any safety valves in place that are 100% secure... So I say, yes, this person can have it… if they’re on a research team with the other person is going to see the data anyways... You have the researchers starting public companies…and then that information that they received under a secure process has now moved from academia out into the public sector…I just don’t see that this is ever going to be really safe. That’s concerning.” CG01

a CP: computer perception.

b Not applicable

### Transparency of Data Sharing

Most participants expressed a desire for greater transparency regarding how CP data are collected, used, and shared. Transparency was valued as a means of maintaining trust and agency, though participants varied in whether transparency alone was sufficient without accompanying control. Participants (6 adolescents and 6 caregivers) sought clarity about what data were collected and when, asking “what information it’s gathering, when it’s gathering it, what it’s tracking” (CG07; see Table 6). Others focused on secondary use, questioning “Where is this going? Who are you sharing it with? What are we doing with this information?” (CG04). Some participants (8 adolescents and 7 caregivers) emphasized the importance of knowing how access was authorized, asking, “Who authorizes that sharing?” CG15; “How is it controlled? What happens if somebody who isn’t supposed to see it, [sees it]?” A16), and how transactions will be tracked (“How are you going to document?” CG11).

**Table 6.** Quotes illustrating varying preferences for transparency in CP^a^ data exchange practices.

Transparency preferences: stated rationales	Adolescent quote	Caregiver quote
Strong desire for transparency		
Desire for transparency of what data CP collects	“I think I would definitely want to know what information is being tracked and how or where the information is going and how it’s being used.” A02	“I’d want to know exactly what information it’s gathering, when it’s gathering it, what it’s tracking.” CG07
If given the chance, they would audit their data transactions frequently	“[I would check] Pretty often I suppose. Mainly because it is a lot about me [that] not a lot of people know about**,** so having a lot of people look at it, I would probably be like, oh, this is out there and let me make sure it’s people I trust and approve of seeing it.” A04 “I’d want to know just what it’s [that data] going to. I wouldn’t like say, no, you can’t use this information… But I would want to know just who’s using it. I would just check up every now and then to see okay. What’s happening with it?” A02	“I would expect there would be an alert, some notification that that is happening. And maybe check it once a week anyway myself, kind of like what I do with my bank accounts, just go check and see if the charges are what I made. That’s something that I would feel comfortable with… [then] I would be able to reach out that much sooner to the research institute and start asking questions.” CG04
Desire for transparent data sharing policy (eg, at time of consent)	“…mainly just with the privacy, but how it’s being stored and who gets to review it and also just the development. If that information is given out early on, then I’ll be like, ’Oh, feel more secure using the tracking and such.’” A16	“I would want to know what they were collecting and for how long. If they’re going to do it for six weeks, I would want to know some assurance that at six weeks it’s no longer collecting data. And then how they were going to protect the information... Who had access to it, I guess. And that they had some steps in place to protect the information.” CG17
Desire for transparency about data protections	“How is it controlled? Who gets to see it? When are they able to see it and do the person who gave the data have control over who gets to see it? And then…what happens if somebody who isn’t supposed to see it sees it and stuff like that.” A16	“[I would want to know] the privacy or the security of that data, that it wouldn’t fall into the wrong hands, misused. I mean, collecting data for me, is wonderful. You get a different picture combined with the actual person’s thoughts, ideas, it’s more meaningful, but it’s more about the security to that, I would always have a concern about.” CG16
Moderate desire for transparency		
Value transparency, but only if accompanied by opportunities for control	“If I’m not able to do anything about it and I see that there’s a company that’s using my data in a way that I don’t want, then I’d rather not know about it. But if there is something that I can do about it, then I’d like to be updated on it and know about it and check it often.” A07	“I don’t think there’s any point in [transparency]…when you know something [that] you can’t do anything about.” CG01
Might have negative reactions to discovering unwanted data transactions	“I’d be pretty weirded out if I didn’t know about it or I wasn’t made aware of it. And I think I’d be pretty upset if it was used by a company to make a product that I don’t support or something like that.” A03	“Well, if they’re getting access to it and I didn’t give them consent to it, I’d be very upset. If I don’t recognize it, I would like to be able to ask somebody who it is. But if it’s just a company that just buys and sells data, no, I would not be very happy about that.” CG02
Moderately interested in transparency and, if given the chance, would only audit their data transactions once in a while	“I think if I looked at [transparency of data transactions] too much, I’d be obsessed and paranoid about it. So I’d try to keep a limit on it.” A03	“I think that would be interesting. I don’t know that I would want it because I want keep track of who all has the information, but **just to kind of see, ‘Oh, this company is using this and they’re getting this out of it,**’ kind of thing. Just out of curiosity, not out of need to know. [I would check] very randomly and probably pretty rarely.” CG20
Low desire for transparency	—^b^	—

a CP: computer perception.

b Not applicable

Additionally, participants (6 caregivers and 2 adolescents) also desired reassurance through transparency of data security processes. For example, specific reassurances included wanting transparency around “how they were going to protect the information” (CG17), along with “how safe and secure the information is” (CG10) and also “some assurance [that] it’s no longer collecting data” when it is physically turned off (CG17). Other participants (3 caregivers and 3 adolescents) shared that they would feel “weirded out” (A07), “upset” (CG09), “disappointed” (CG16), “angry” (CG07), and “violated” (A16) if shown transactions with their data that they did not explicitly consent to.

Other participants (n=4) expressed that transparency without the ability to intervene would be frustrating or distressing, noting they would “rather not know” if no action were possible (A03). As one caregiver put it, “I don’t think there’s any point… when you know something [that] you can’t do anything about” (CG03). Another adolescent participant reasoned that “[they’d] rather not know about it” if they could not act on the knowledge (A03).

## Discussion

### Participant Perspectives Toward Current Data Protections

A majority of adolescents and caregivers view CP data, that is, data collected through wearable digital sensors, as sensitive information, characterizing clinical assessments using CP as “invasive” (a term used by multiple participants). Many felt that such data could enable insights into patients’ emotions and overall well-being. This is, of course, the clinical rationale for collecting these data; however, many respondents expressed privacy concerns, particularly around the potential for stigma or discrimination in cases of unintended or unwanted disclosure, given that participation was connected to their mental health condition. Some respondents stated that CP data thus deserve as much as or greater protection than other personal health data, which should be kept private and deidentified before sharing, as previous studies have found. Consistent with other research findings [50-52], participants from our study said they would feel distressed upon discovering that their data were being used in ways they did not explicitly consent to, especially for commercial purposes. Their concerns are broadly echoed in the literature around CP technologies [53-55], as well as for nonhealth-related data [31,56-59].

Importantly, however, most participants believe that CP data already receives high levels of protection and security. Many stated they highly trust their clinicians and hospitals to protect CP data once collected and expressed confidence that CP data fall under HIPAA protections as with other medical information. Others said they would seek “reassurance” that this is the case, revealing that they consider HIPAA protection to be the default. Respondents were generally unaware that many, if not most, forms of CP data are not classified as protected health information (PHI). CP data often falls outside traditional health privacy protections for two reasons. First, many tools are developed by companies that are not covered health care entities under HIPAA. The data generated by these technology products is instead subject to other data protection laws at the federal level (eg, Gramm-Leach-Bliley Act, Federal Trade Commission Act, and Family Educational Rights and Privacy Act) and state level (eg, California Consumer Privacy Act, California Privacy Rights Act, and Virginia Consumer Data Protection Act). However, critics [60,61] have characterized these as a “patchwork” of data protections that are too specific to certain data types or entities or contain various loopholes that create data protection gaps for citizens, especially for consumer-facing health apps and wearables.

Second, many of these data types (eg, GPS, accelerometry, call logs, message logs, or content) are not directly related to a patient’s health or diagnostic status, even if they may in some cases be collected in service of clinical objectives. As other scholars [18] have noted, these forms of NTH data occupy an ambiguous or gray area in health data protections, even more so when they are uncoupled from the 18 identifiers that define HIPAA-protected PHI, as HIPAA does not cover deidentified information. This gap in legal protections for deidentified data becomes increasingly problematic as the ability to reidentify data grows, or when certain types of data, such as facial expressions and voice, are increasingly hard to deidentify. These concerns around privacy have been widely debated in the context of insurance, genomics, and other domains of data governance [62]. A burgeoning industry devoted to reidentifying personal data using machine learning technologies (eg, for marketing purposes or other forms of profiling) increasingly enables unwanted disclosure of personal data. The potential for reidentification is especially high for NTH that is subject to data exchange decisions that are outside of patients’ (and even many physicians’ and researchers’) control.

An important takeaway from our findings in this context is that most patients and caregivers erroneously believe that HIPAA protections guard them from unwanted disclosure of their PHI and may not recognize the limitations of existing data protection frameworks [63]. Importantly, participants rarely differentiated between research and clinical data-sharing frameworks, despite these contexts being governed by different standards for consent, oversight, and commercial use. This lack of differentiation reflects the reality that CP data are often collected and reused across translational pipelines in ways that obscure whether data are governed by research ethics approvals, clinical privacy regimes, or commercial data practices. This ambiguity contributes to epistemic vulnerability and complicates their ability to make informed decisions about whether to consent to the use of CP technologies in clinical care or research. When individuals later learn that protections are weaker or more fragmented than assumed, this mismatch can undermine trust, reduce willingness to share data, and negatively affect adoption and sustained engagement. These findings echo broader public concerns about digital privacy beyond health care, where individuals often misunderstand the extent of their data protections [64-66]. Similar trends exist in consumer data ecosystems, particularly in digital marketing, where data collection occurs without explicit user awareness [30]. To bridge the gap between perceived and actual protections, it is critical to educate individuals about the limitations of data protections across these domains and the circumstances under which their data may be used without their consent. Education can help to promote greater reflection about potential threats to the security and privacy of the information that individuals agree to have collected and managed. The use of decision aids and other decision support approaches (eg, shared decision making) offers frameworks to ensure patients and caregivers receive sufficient risk/benefit information to engage in informed consent. However, as we discuss below (see “Clearly Communicating (and Knowing) Risk”), certain factors limit the efficacy of education as a primary approach to achieving informed consent.

### Challenges to Patient Education and Informed Consent About CP Data Collection

#### Clearly Communicating (and Knowing) Risk

Several factors challenge the feasibility of effectively educating participants about health data protection and data-sharing. One major challenge is the elusive nature of data sharing. Hospitals, research institutes, data brokers, electronic health record companies, and other entities that comprise the HIE market often engage in transactions that are not made public or fully transparent to individuals who are the subjects of that data [67]. This lack of transparency endures despite findings, including those from the present study, that most individuals desire greater visibility into the nature and extent of secondary data sharing. Similar demands for transparency and accountability have been observed in nonhealth contexts, such as media personalization and digital marketing [33]. Other studies likewise suggest that people—especially young people—want an active, participatory role in decision-making about data sharing [50]. While most (deidentified) data exchanges are permitted under HIPAA, their notorious opacity (regarding with whom, why, how often, and for what profit) complicates the ability of researchers and clinicians to adequately inform participants about the risks associated with participating in research studies that capture sensitive data. Often, clinicians and researchers themselves are unaware of the nature and extent of potential risk in ways that would enable them to clearly communicate them to research participants, as required by most institutional review boards. Greater empirical insights are urgently needed into the impacts of collecting these data in practice. For example, what percentage of these data are deidentified and exchanged only to be reidentified? What percentage of these reidentified data, in turn, lead to patient harms, and what kinds of harm (eg, dignity-related, financial, physical, emotional, social, political, etc)? Answering such questions requires forensic analysis that traces data from its source as it moves across the HIE landscape. Such an endeavor could be facilitated by various technologies that enable transparent tracking of provenance and transaction histories, as we have argued elsewhere [68,69].

Currently, however, HIE transactions remain critically understudied. While new federal legislation has been proposed (eg, American Privacy Rights Act, American Data Privacy Protection Act, and Consumer Online Privacy Rights Act) to address consumer privacy concerns, to ensure greater control over personal information, and to establish robust enforcement mechanisms to hold companies accountable for data sharing practices, federal funding for empirical research into the nature and extent of secondary data exchange is still lacking. Critics argue that HIE remains largely unaddressed and unregulated due to fragmented state and federal laws [70,71], lobbying by powerful electronic health record companies seeking to maintain market dominance, and fears that regulation may stifle innovation in bioinformatics. The result is an enduring unawareness among most individuals, including experts, about what happens to their health (and other types of) data once collected.

One remedy might be to mandate greater visibility into health data transactions, such that patients and research participants can follow who sees their data, how often, and for what purposes (eg, data sharing between researchers, data exchange for profit, etc). We have described elsewhere some computational avenues (eg, distributed ledger technologies) for enhancing the transparency and auditability of data exchanges [68,69]. However, even if these mechanisms are put in place, our findings suggest that many participants have mixed feelings about being able to see transactions involving their health information; some express enthusiasm, while others point out that such transparency would only frustrate them if unaccompanied by the ability to intervene in or control those transactions. To date, limited mechanisms exist to empower such control. Approaches such as dynamic consent, which enable participants to continuously monitor, manage, and update their consent preferences over time, may be appealing to individuals who want to actively track transactions involving their data (in this study, three-quarters of our participants). Notably, dynamic consent mechanisms are still not common in clinical research outside of certain contexts, for example, genomic or long-term biobanking, where data may be repurposed numerous times for multiple research agendas. In most areas of research, traditional forms of consent prevail, particularly “broad” consent where patients consent once at the start of a study or treatment and cannot modify stated permissions thereafter. Despite affording participants almost no insight into secondary use applications, broad consent enjoys widespread support from the scientific community [72,73], as it can help to facilitate data sharing and drive scientific progress. However, it requires high trust in data controllers to make decisions that align with data subjects’ preferences. Our findings show that trust is indeed high among most research participants, but perhaps it should not be, not necessarily because data controllers do not act in good faith when stewarding participant data, but because the current legal landscape offers limited protection for health and NTH data that may enter into a cascade of secondary data exchanges that are difficult for primary data controllers to forecast, trace, or control.

#### Avoiding Undue Aversion and Consent Refusal

Determining the appropriate level of risk information for clinician researchers to communicate to participants is not only challenged by a lack of clarity about the extent and nature of harms but also by researchers’ awareness that highlighting such (uncertain) risks may generate risk aversion and lead to consent refusal. The inability to assuage potential participants’ concerns is exacerbated by the difficulty of auditing or assessing when unwanted disclosure and associated harms do occur. Individuals may be reluctant to consent to secondary uses of their data if they fear potential harms will go unnoticed and continue to perpetuate indefinitely. Balancing disclosure of potential risks while encouraging participation remains a complex challenge. Improved ethical frameworks are needed to communicate uncertain risk without “scaring off” potential research participants who may benefit from integrating CP into their clinical care or contribute significantly to scientific discovery and research. For example, in the context of genomic sequencing, Koplin et al [74] argue that appropriately informed consent may be a fitting alternative to fully informed consent in cases where risks are uncertain. Koplin et al [74] argue that focusing consent procedures on satisfying four morally important goals that informed consent is meant to achieve, including (1) respect for personal autonomy, (2) promotion of autonomy and (3) well‐being, and (4) preservation of trust in medicine, may help individuals to make decisions that align with their values, even without a full picture of potential risks. Further work is needed to explore how each of these goals may be achieved in the context of participant consent to CP data collection. For example, what mechanisms may be put in place to ensure autonomy over decisions to share data within and/or beyond a participant’s clinical team? What information is needed to clearly convey the potential benefit of CP data collection for participants’ well-being, especially given that most CP technologies remain investigational? These questions deserve greater attention as CP and mHealth technologies continue to gain traction in health care and research.

### Pathways Forward

Given these uncertainties, it remains unclear how clinicians and researchers should proceed with informed consent. On the one hand, they are ethically obligated to communicate risks associated with the use of CP in research or care, but they themselves lack sufficient insights into these risks. Further, they must be careful not to over- or understate risks, balancing awareness against the potential to affect individuals’ willingness to participate in research or integrate technologies that may ultimately benefit patients. Greater visibility into the nature and extent of transactions may only partially solve the problem of informed consent, as it may lead to distress if accompanied by opportunities for intervention or control. However, data controllers (ranging from researchers, institutions, companies, and data brokers) currently have little incentive to offer such control if legal mandates are not in place (and there is no indication they will be anytime soon). A key arena to watch for developments around such mandates is the newly created federal Trusted Exchange Framework and Common Agreement (TEFCA; 2024), which aims to facilitate nationwide, query-based data sharing and to improve patient access to health information as mandated by the 21st Century Cures Act (2016) [75]. Mandel et al [76] recently argued that three principles should be put in place by the time TEFCA launches, including that patients can (1) query for data about themselves, (2) know when their data are queried and shared, and (3) configure what is shared about them. TEFCA launched in 2024, and so far, while it does appear to support patient access to their own health information, TEFCA does neither specifically mandate that patients be notified each time their data are queried or shared, nor do all constituent health information networks offer the capacity for patient-configurable data-sharing controls. Research to identify and validate procedures and technologies that may provide the necessary infrastructure for such controls should be prioritized at the federal level to evolve alongside emerging data privacy legislation.

### Limitations

This study has several limitations. First, our qualitative sample was drawn from adolescents and caregivers already enrolled in a research study embedded within a clinical research setting. Participants may thus have higher baseline trust in clinicians and institutions than individuals engaging with direct-to-consumer digital health tools, potentially limiting generalizability to other populations or contexts. Second, participants’ perspectives reflect experiences at the intersection of research and clinical care; while this overlap is increasingly common for CP technologies, findings may not fully capture attitudes in purely clinical or purely commercial settings. Third, although the primer video was designed to ensure a shared baseline understanding of CP technologies, it may have influenced participants’ framing of risks and benefits. Finally, as with all qualitative research, our findings are not intended to quantify prevalence or establish causal relationships, but rather to identify salient themes and ethical concerns that warrant further empirical investigation.

### Conclusions

Our findings highlight the need for clearer communication, stronger data governance, and consent models that better reflect how digital health data are used in practice. These data hold strong potential to provide ecologically valid insights into patients’ health conditions beyond the clinic, but also pose significant privacy risks that remain understudied. While clinicians and researchers have a unique responsibility to safeguard participant and patient data, they cannot effectively do so without a better understanding about the nature and extent of these risks or how to effectively mitigate them. To promote greater visibility, understanding, and trust (when justified) in digital tools that have strong promise to promote patient health, we advocate for five key strategies, including: (1) educating patients on the limitations of existing data protections; (2) conducting targeted research, including forensic analyses, into secondary data exchanges and identifying privacy breaches or reidentification risks; (3) enacting regulations that mandate greater transparency in health data transactions; (4) implementing computational mechanisms, such as distributed ledger technologies, to enhance data traceability and auditability; and (5) adopting dynamic consent models that allow patients to continuously manage and update their consent preferences. These strategies may help to better balance the promise of CP technologies and insights from wearables and mHealth apps with the ethical imperative to protect patient privacy, fostering greater trust and responsible innovation in digital health care.

## Supplementary material

10.2196/78788 Multimedia Appendix 1 Adolescent interview guide.

10.2196/78788 Multimedia Appendix 2 Caregiver interview guide.

10.2196/78788 Multimedia Appendix 3 Explainer video of the ethics of perceptive computing in health care study (EPICH).

10.2196/78788 Checklist 1 COREQ Checklist.

## Abbreviations

CP: computer perception
GDPR: General Data Protection Regulation
HIE: health information exchange
HIPAA: Health Insurance Portability and Accountability Act
mHealth: mobile health
NTH: nontraditional health
PHI: personal health information
RA: research assistant
TEFCA: Trusted Exchange Framework and Common Agreement
