# Supplementary material for: Perceived Sensitivity of Sensor-Based Digital Health Data: Qualitative Interview Study
Source: JMIR Mhealth Uhealth. 2026 Jul 8;14:e78788. doi: 10.2196/78788 (PMC13345348; doi:10.2196/78788)
Supplement: Multimedia Appendix 1 [file mhealth-v14-e78788-s001.docx]

|  | **Question** | **Notes** |
| --- | --- | --- |
| **1** | 1. **Introductions** 2. **Ask about consent form**   Today, I’m going to ask you some questions about the t**ypes of information that you provided** during your participation in the ACES project -- and it’s totally okay if you haven’t thought about any of these topics before. **We are just looking for your initial reactions** and all of your input is **valuable**! |  |
| **2** | Do you remember the video from your last interview, explaining **some of the data types that were collected in the ACES project?**  (*If no*: Show it)  *If yes*: **Would you like to review it again**, just to make sure you understand the types of data we’re going to be talking about today? (or are you good)?   1. ***START RECORDING*** | **After Slide 3:** Do you have any questions about what computer perception is?    **After Slide 5:** Did you understand how these technologies can be used inside the doctor’s office?    **After Slide 7:** Do you have any questions about how detecting these sources of information could tell your doctors how you are feeling outside of the clinic?    **After Slide 11:** Did you have any questions about the types of data being collected and why it might be helpful?    **After Slide 14:** Were there any questions you have about any information discussed in the video or how this might impact your usual doctor’s appointments? |
| **3** | When you were having these particular types of data collected, for example using the cameras and microphones in the research lab, **were you thinking at any point about what happens to the data after it is collected?**    Follow-up: (Now that I’ve mentioned it) What kinds of **questions** or **concerns** did you have **about where this particular data goes, who sees it, or where it’s stored**?    *(Probe: For example, how social media platforms have* ***different levels of permanence****, like Snapchat messages that* ***disappear*** *after being opened or stories that only last for a day --* ***How permanent do you view*** *the type of* ***data that you provided****?)* |  |
| **4** | **Sometimes** this data is collected **in the clinic**, like you experienced, and **sometimes** they’re collected using **wearables** and other devices **outside of the clinic**, like you saw described in the video.    **How do you feel about certain information** – like about your emotional ups and downs or behaviors – being **automatically collected** “in the background” using these technologies as you go about your day?    Follow-up: **Anything that interests** you or maybe **makes you feel uncomfortable** about it? |  |
| **5** | How much do you feel that **this particular type** of data (about your voice, facial expressions, and gestures) **should be kept private**? Why?    Follow-up: **How much do you care** about **whether or not anyone can access** the kinds of data you provided for the research you just participated in?  Would you say you care: **Very much, a little, or not at all**? |  |
| **6** | Some people say **young people** **don't care about privacy**. In your experience, is that true?  Follow-up: Can you remember a **time or situation** – at school or online, for example – **where you were tracked or monitored** in a way that **made you uncomfortable**? |  |
| **7** | When you go to the hospital, sometimes you’re asked to give some kind of biological sample (like a blood or urine sample) or even a genetic sample.  The **data samples you provided** for the research project you just participated in were a bit **different** -- **cameras** recorded your facial expressions and gestures, and **microphones** recorded different aspects of your voice.    Do you feel that there is **anything particularly different about these types of data**, in terms of **what they might reveal about you**, than say, a blood test? Can you elaborate?    Follow-up: Do you think that this data **should have the same level of protection** as, say, biological data, like a blood or urine sample, or should it receive **more** protection, **less** protection? **Why**?  *If they ask, well why would it be any different?:*  *Some people might feel like information about your emotions or behaviors is more sensitive or* ***might reveal more*** *about you,* ***personally****, than a biological blood sample, for example.*  *Or some people feel that there might be stigma around “****negative****” emotions or behaviors, like if you were often feeling angry or depressed or anxious… S****ome people might not*** *want others to* ***know*** *that.*  *Do* ***YOU*** *feel that psychological and* ***behavioral*** *information is any different or should receive any* ***different protections*** *than say, biological or even genetic data?* |  |
| **8** | Sometimes when data is collected, **you're asked if you would allow** your data to be used in other research projects. That means that the data could be used in **future research** that might not have even been thought of yet.  **How do you feel** about **allowing this particular kind of health data – about your behaviors and emotions,** in **future research?** |  |
| **9** | **How important** is it for you **to know** the specifics about the **purpose of the research** project and/or **who the researchers are** that are conducting it before deciding to **allow** access to your data?  *Probe: For example, in terms of “who” -- some people might only be okay sharing their data with researchers but not with private companies.*  *Or in terms of “purpose” – some people might want to share their data for purposes they care about, and not for those that aren’t directly relevant to them personally (like alzheimer’s research) or that they might not agree with (like stem cell research).*  *Is it important for you to* ***know*** *who you’d be sharing your data with or for what purposes?* |  |
| **10** | Is there anyone who you think **SHOULD** be able to see or use **this specific type of data** about **you**, **other than** your parents, doctors and the research team analyzing it?    *(Probe: Like other researchers who are not part of this research team; or like companies interested in developing products using your data)* |  |
| **11** | Is there anyone you **would NOT want to be able to see or access** this specific type of data about you?    *(Probe: Like other researchers, companies, insurance companies, future employers)* |  |
| **12** | So, switching gears, the laws that exist in the U.S. right now (you might have heard of “HIPAA”) **allow** doctors and researchers to **share** patient data as long as it is **de-identified.** That used to be an effective way to protect data. But **now** there are lots of ways to use **OTHER** data out there on the internet **to RE-identify** you. So, **even de-identified data that is shared might not be as protected as it used to be**. Does that make sense?  I want talk about the **specific transactions** or sharing that happens with **your specific personal health** data with certain entities, like other research teams, hospitals, companies, and people who buy and sell your data. Does that make sense?  How would you **react** if your doctor told you that **there was an app that would allow you to see who accesses or uses** the health information that you share (like your emotional ups and downs or behaviors), **how they use it, and for what purposes**?  Would this app **interest** you? **Why** or **why not**? |  |
| **13** | **If you saw** on this app that **your data was being accessed** by people, institutions or companies you've never heard of, **what would be your reaction**?    Follow-up: Is there **anything you’d want to know about them** **to decide** whether or not you’d be “ok” with sharing with them? How would you figure that out? What would you look for? |  |
| **14** | **How much** do you think **you'd take a moment to take a look at who’s accessing your data**? Hardly at all, a little, or pretty often? And **why**?    *(Probe: How curious would you be? / How important would it be to you?)* |  |
| **15** | Would you be **interested in** being able to **see how your data was contributing to science** like helping to **help cure diseases** or **improve treatments?**    Follow up: **How much** do you think **you’d use or open an app** that allowed you to track your **contributions** to science or health research? Hardly at all, a little, or pretty often? And **why**? |  |
| **16** | Would you be **interested being able to see how companies are using your data** to generate **profits** or to help with **marketing**?    Follow up: **How much** do you think **you’d use or open an app** that allowed you to **track** how **companies** are using your data? Hardly at all, a little, or pretty often? And **why**? |  |
| **17** | Which option would you prefer: Being able to **access this information about how your data is being used or shared anytime** you want or, instead, **getting a report every 6 months** (or so) of how your **data was being used or shared?** |  |
| **18** | **Who** do you think **should control or decide** who can see your personal health data?    Follow up: Should it be **you**? Should it be **you and a parent**? Should it be your doctor, or the researchers who are collecting the data? Everyone? |  |
| **19** | Would **you** want **someone to help** you make these decisions about who to share your data with? (If yes: who?)    Follow-up: Would you trust researchers, doctors or other members of your community **to make decisions for you** (on your behalf) about who **can** and **cannot access** your data, acting in your best interest? |  |
| **20** | Would you be interested in **actually choosing and controlling**who has permission to see your data? **Why** or **why not**? |  |
| **21** | Would the **ability to see or control** who accesses your data **influence your** **willingness to share** your personal health information in general? **Why?** |  |
| **22** | Would you want the **ability to change your mind** in the future about who you share **your** data with, maybe as you get older? Or would you **prefer to just state your preferences once** and be done with it? |  |
| **23** | Do you think patients should get some kind of **compensation** or **reward for sharing their personal health data with** people, institutes or companies that use those data to develop services or products?    Follow up: If so, **what kind of rewards**? Can you think of an **example**? |  |
| **24** | Now that we've talked about your data sharing preferences, do you feel **any differently** about your healthcare data and privacy?    What sort of **questions** or **comments** are you left with?    Anything you’d like to **talk about related to data protections** or privacy that I **haven’t asked** about? |  |
